# The Endogenous Nitric Oxide Mediates Selenium-Induced Phytotoxicity by Promoting ROS Generation in *Brassica rapa*

**DOI:** 10.1371/journal.pone.0110901

**Published:** 2014-10-21

**Authors:** Yi Chen, Hai-Zhen Mo, Liang-Bin Hu, You-Qin Li, Jian Chen, Li-Fei Yang

**Affiliations:** 1 College of Horticulture, Nanjing Agricultural University, Nanjing, China; 2 Institute of Food Quality and Safety, Jiangsu Academy of Agricultural Sciences, Nanjing, China; 3 Department of Food Science, Henan Institute of Science and Technology, Xinxiang, Henan Province, China; University of Missouri-Kansas City, United States of America

## Abstract

Selenium (Se) is suggested as an emerging pollutant in agricultural environment because of the increasing anthropogenic release of Se, which in turn results in phytotoxicity. The most common consequence of Se-induced toxicity in plants is oxidative injury, but how Se induces reactive oxygen species (ROS) burst remains unclear. In this work, histofluorescent staining was applied to monitor the dynamics of ROS and nitric oxide (NO) in the root of *Brassica rapa* under Se(IV) stress. Se(IV)-induced faster accumulation of NO than ROS. Both NO and ROS accumulation were positively correlated with Se(IV)-induced inhibition of root growth. The NO accumulation was nitrate reductase (NR)- and nitric oxide synthase (NOS)-dependent while ROS accumulation was NADPH oxidase-dependent. The removal of NO by NR inhibitor, NOS inhibitor, and NO scavenger could alleviate Se(IV)-induced expression of *Br_Rbohs* coding for NADPH oxidase and the following ROS accumulation in roots, which further resulted in the amelioration of Se(IV)-induced oxidative injury and growth inhibition. Thus, we proposed that the endogenous NO played a toxic role in *B. rapa* under Se(IV) stress by triggering ROS burst. Such findings can be used to evaluate the toxic effects of Se contamination on crop plants.

## Introduction

Selenium (Se) is an essential and beneficial micronutrient for plants [1]. The important roles of Se in intrinsic physiological process has been confirmed by the identification of Se-containing proteins in plant cells [2], [3]. The moderate supplement of Se is able to protect agricultural plants from multiple environmental stresses [4]–[10]. However, Se is becoming an emerging health hazards in a global scale because of the dramatically anthropogenic release of Se to the environment [11]. The excessive Se in agricultural environment poses potential threat to the growth of crop plants by causing phytotoxicity [12]–[14]. The lack of knowledge on the mechanism of Se-induced phytotoxicity limits the evaluation of the toxic effect of Se on crop plants. Several recent studies have suggested that the excessive Se could induce oxidative stress in plants by triggering the production of reactive oxygen species (ROS) [15]–[17]. However, how Se induces ROS generation and the subsequent oxidative injury in plants remains unclear. NADPH oxidase coding by *Rbohs* (*respiratory burst oxidative homologues*) has been suggested as a major source for ROS generation in plants under both biotic and abiotic stresses [18]. The *Rboh* family contains 10 annotated or putative *Rboh* genes (*RbohA-J*) in *Arabidopsis* genome [19]. The stimulated NADPH oxidase activity in plants has been associated with heavy metal stress [20]–[22], but the comprehensive regulation of different *Rboh* members by heavy metals (including Se) is hardly known.

Nitric oxide (NO) is a pivotal signalling molecule in regulating the key physiological processes during plant development and stress responses [23]. Nitrate reductase (NR) and nitric oxide synthase (NOS) are the two major enzymatic sources for the endogenous production of NO in plants [24]. *NOS* gene has not been identified from plants, but the activity of NOS has been successfully detected in plants [25], [26]. There are other NO producing pathways including aerobic NO formation based on hydroxylamines or polyamines and anoxic NO formation based on deoxygenated hemeproteins, but the exact molecular mechanisms of these NO-producing pathways are still highly speculative [27]. The protective role of NO in plants against metal-induced oxidative stress has been well characterized [28]–[33], but recent studies have suggested that NO could also contribute to phytotoxicity induced by heavy metals, such as cadmium (Cd) [34]–[36], arsenic (As) [37], and aluminium (Al) [38]. NO is proposed to be a stimulator of hydrogen peroxide (H_2_O_2_) in Cd-induced cell death in Arabidopsis [34]. However, the mechanism of endogenous NO-mediated ROS generation and oxidative stress is currently not understood in plants under heavy metal stress.

In this study, the relationship between Se-induced phytotoxicity and NADPH oxidase-governed ROS generation was investigated in the root of *Brassica rapa*. The effect of Se stress on the production of endogenous NO from different sources was studied *in vivo* by fluorescent microscopy. To get deeper insights into the interplay between the endogenous NO and Se-induced oxidative stress in *B. rapa*, the involvement of the endogenous NO on the differential expression of *Rboh* genes, oxidative injury, and growth inhibition was investigated. All of these results are very important to help our understanding for the toxic role of NO in plants under Se stress, which will assist to the evaluation of the environmental risk of Se to crop growth.

## Materials and Methods

### Plant culture and treatment

Seeds of wild type *B. rapa* (LvLing) were sterilized with 1 % NaClO for 10 min, then rinsed several times with distilled water and germinated for 1 day in the dark on the floating plastic nets. After germination, young seedlings were transferred to Petri dish containing various treatment solution in a chamber with a photosynthetic active radiation of 200 µmol/m^2^/s, a photoperiod of 12 h, and the temperature at 25±1°C.

Seedling roots were exposed to Na_2_SeO_3_ (sodium selenite) with different concentrations (0.03–0.46 mM) for various treatment time (0–72 h). DPI (diphenylene iodonium) at 10 µM, PY (pyridine) at 5 mM, and IMZ (imidazole) at 0.5 mM were applied as NADPH oxidase inhibitors [39], [40]. Tungstate (Na_2_WO_4_) at 30 µM and _L_-NMMA (N^G^-Monomethyl-L-arginine) at 200 µM were applied as NR inhibitor and NOS inhibitor, respectively [41], [42]. The 0.1 mM of cPTIO [2-(4-carboxy-2-phenyl)-4,4,5,5-tetramethylinidazoline-1-oxyl-3-oxide] was applied as NO scavenger [43]. SNP (sodium nitroprusside) at 0.25 mM was applied as NO donor [43]. The treatment solution is composed of different chemicals mentioned above according to different experimental design. After treatment, the roots were washed with distilled water for physiological, histochemical, and biochemical analysis.

### Histochemical analysis

Intracellular ROS was visualized using DCFH-DA (2′,7′-dichlorofluorescein diacetate) fluorescent probe described by Foreman et al [44]. The roots of seedlings were incubated in 1 µM of DCFH-DA at 25°C for 20 min. Then the roots were rinsed with distilled water for three times followed by the visualization (excitation 488 nm and emission 525 nm) with a fluorescence microscope (ECLIPSE, TE2000-S, Nikon).

Intracellular NO was visualized using DAF-FM DA (3-Amino, 4-aminomethyl-2′,7′- difluorescein, diacetate) fluorescent probe described by Guo et al [45]. The roots of seedlings were incubated in 20 mM of Hepes-NaOH (pH 7.5) buffer solution containing 15 µM of DAF-FM DA at 25°C for 15 min. Then the roots were rinsed with distilled water for three times followed by the visualization (excitation 490 nm and emission 525 nm) with a fluorescence microscope (ECLIPSE, TE2000-S, Nikon).

O_2_^• **−**^ in shoots was visually detected by using NBT (nitro blue tetrazolium) described by Frahry and Schopfer [46]. The roots of seedlings after treatment were transferred to 10 mM Na-citrate buffer (pH 6.0) containing 6 mM NBT under light at 25°C for 20 min, and then the roots were rinsed with distilled water for three times, which allowed the dark blue insoluble formazan compound (by reaction of NBT with O_2_^• **−**^) inside of roots to be clearly visualized and photographed.

Histochemical detection of lipid peroxidation was achieved by using Schiff′s regent as described by Wang and Yang [28]. The roots of seedlings after treatment were incubated in Schiff′s regent for 20 min. Then the stained roots were rinsed with a solution containing 0.5% (w/v) K_2_S_2_O_5_ (prepared in 0.05 M of HCl) until the root colour became light red. After that, the roots were photographed using a digital camera.

Histochemical detection of loss of plasma membrane integrity was performed by using Evans blue as described by Yamamoto et al [47]. The roots of seedlings after treatment were incubated in 5 ml of 0.025% Evans blue solutions (w/v) for 20 min. After that, the roots were rinsed with distilled water for three times followed by photographed using a digital camera.

### Screening and analysis of *Br_Rbohs* from the genome of *B. rapa*

The sequences of *AtRbohA-J* from *Arabidopsis* were used as baits for BLAST research in the genome of *B. rapa* from BRAD (*Brassica* database) (http://brassicadb.org/brad/index.php). The obtained sequences were retrieved and analyzed. The phylogenetic trees were constructed using the maximum likelihood method in MEGA 5.2. The multialignment of amino acid sequences was performed with ClustalX 2.0 and DNAMAN 5.2.2. Protein structure prediction was performed on SMART (http://smart.embl-heidelberg.de/).

The DNA sequences with the length of 2 kb were retrieved from the upstream of *Br_Rbohs* in *B. rapa* for promoter analysis. The sequence between the start of target gene and the end of its upstream gene was obtained for promoter analysis if the length of this sequence was less than 2 kb. The *cis*-elements in the retrieved promoter regions were analyzed using online tool PLACE (http://www.dna.affrc.go.jp/PLACE/signalscan.html).

### Transcript analysis

Total RNA was extracted from root tissues using Trizol (Invitrogen) according to the manufacturer's instructions. Reverse transcription was performed at 42°C in 25 µl reaction mixture including 3 µg of RNA, 0.5 µg of oligo (dT) primers, 12.5 nmol of dNTPs, 20 units of RNase inhibitor and 200 units of M-MLV. The first cDNA was used as a template for polymerase chain amplification and to analyse the transcripts of genes by using real-time quantitative reverse transcription-polymerase chain reaction (qRT-PCR) (Applied Biosystems 7500 Fast Real-Time PCR System, LifeTechnologiesTM). The primers designed for the amplification of genes are listed in Table S1.

### Determination of enzyme activities

About 0.05 g of fresh root tissue was homogenized in 1.5 ml of ice-cooled phosphate buffer (50 mM, pH 7.0, containing 1 mM ethylenediamine tetra-acetic acid (EDTA) and 1% w/v insoluble polyvinylpyrrolidone). The homogenate was centrifuged at 15,000 g for 10 min at 4°C. The supernatant was used as the crude extract for the assay of enzyme activities.

The activity of NR was determined as described by Xiong et al [48]. Enzyme extract was added to 0.4 mg/L of pre-warmed assay buffer containing 100 mM of HEPES-KOH (pH 7.5), 5 mM of KNO_3_, and 0.25 mM of NADH. The mixed solution reacted at 30°C for 60 min, and then the reaction was stopped by adding Zn-acetate. The absorbance of produced nitrite was measured at 540 nm by adding 1 mg/L of 1% sulfanilamide in 3 M HCl plus 1 mg/L of 0.2% N-(1-naphthyl) ethylenediamine. A standard curve of NO_2_^−^ in the range 0–2 µg was prepared to convert the measured absorbance to concentration.

The NOS activity was determined by NOS colorimetric kit as described by Li et al [49]. NOS activity was spectrophotometrically measured as the capacity of catalysing NO production from _L_-arginine, which is based on the oxidation of oxyhaemoglobin to methaemoglobin by NO. One unit was defined as generating 1 nmol of NO per minute at 37°C per microgram fresh weight.

### Statistical analysis

Each result was presented as the mean ± standard deviation (SD) of at least three replicated measurements. The significant differences between treatments were statistically evaluated by SD and one-way analysis of variance (ANOVA) using SPSS 2.0. The data between two specific different treatments were compared statistically by ANOVA, followed by F-test if the ANOVA result is significant at *P*<0.05. For multiple comparison analysis, least significant difference test (LSD) was performed on all data following ANOVA tests to test for significant (*P*<0.05) differences among different treatments.

## Results

### Se(IV) induced growth inhibition and oxidative injury in the roots of *B. rapa*

**Figure 1:**
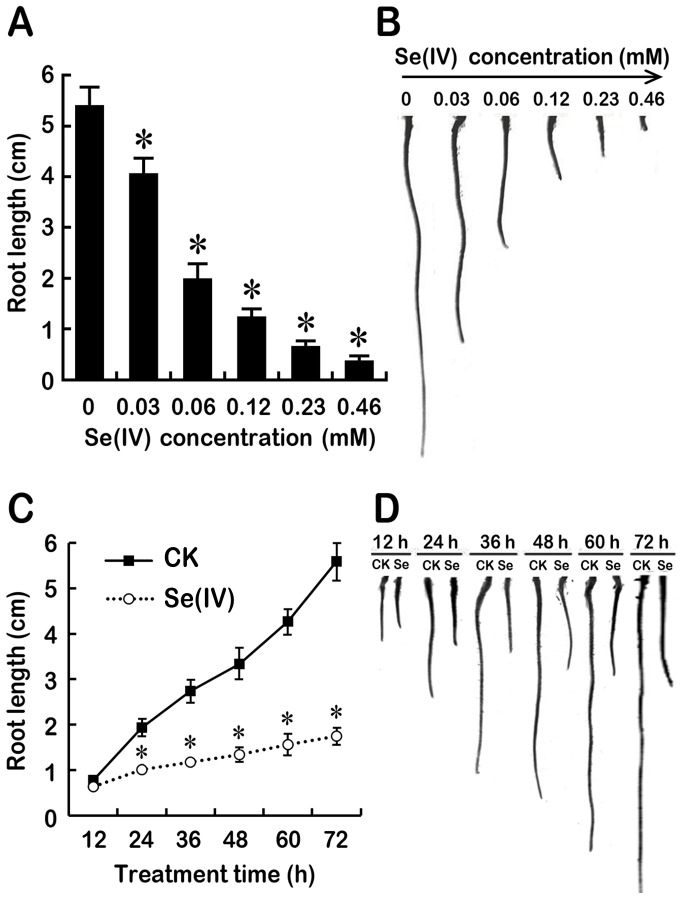
Effect of Se(IV) on the growth of *B. rapa* root. (A–B) The roots of seedlings were exposed to 0, 0.03, 0.06, 0.12, 0.23, and 0.46 mM of Se(IV) solution for 72 h. After that, the root length were measured (A). The images of roots were photographed (B). (C–D) The root length and root image were obtained when the roots of seedlings were exposed to 0.06 mM of Se(IV) solution for 12, 24, 36, 48, 60, and 72 h, respectively. *Asterisk* indicates that mean values of three replicates are significantly different between the treatments of Se(IV) and the control group (CK) (*P*<0.05).

Treatment with Se(IV) inhibited the root growth *B.rapa* in a dose-dependent manner. The roots were exposed to 0–0.46 mM of Se(IV) for 72 h. Compared to the control, the root length decreased by almost 50% at 0.06 mM Se(IV) level (Figure 1A, B). Therefore, 0.06 mM of Se(IV) was used for the further experiments. In a time-course experiment, the root growth was inhibited significantly after treatment with 0.06 mM of Se(IV) for 24 h. Se(IV) at 0.06 mM showed the continuous inhibitory effect on root growth after treatment for 24–72 h (Figure 1C, D).

**Figure 2:**
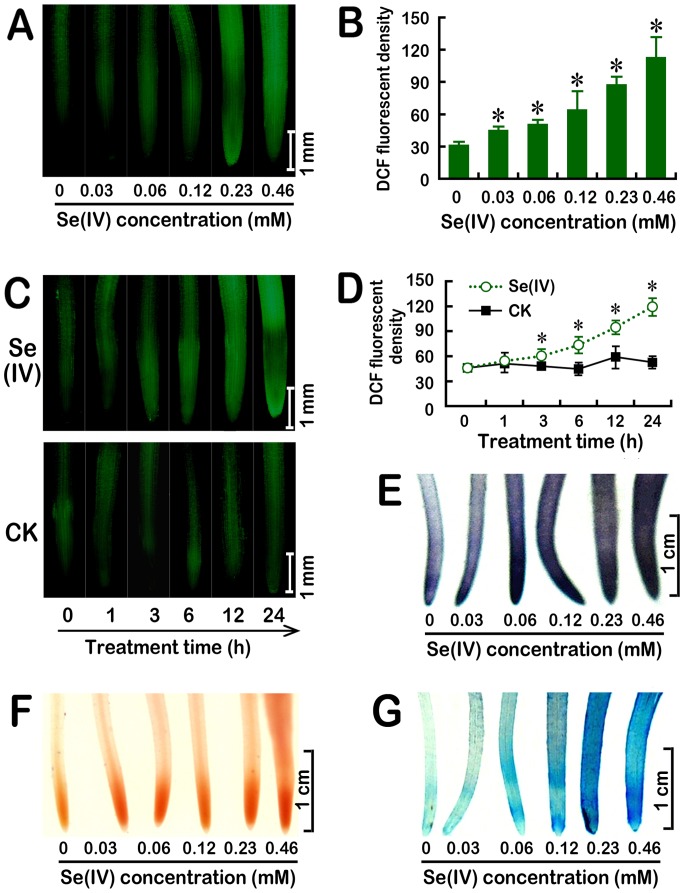
Effect of Se(IV) on endogenous total ROS (A–D), endogenous O_2_^• −^ (E), lipid peroxidation (F), and the loss of plasma membrane integrity (G) in the roots of *B. rapa*. The roots of seedlings were exposed to 0, 0.03, 0.06, 0.12, 0.23, and 0.46 mM of Se(IV) solution for 48 h. Afterwards, the roots were loaded with DCFH-DA for 15 min and immediately photographed (A). The relative DCF fluorescent density in roots was estimated (B). (C–D) The image and density of DCF fluorescence were obtained when the roots of seedlings were exposed to 0.06 mM of Se(IV) solution for 0, 1, 3, 6, 12, and 24 h, respectively. The roots of seedlings were exposed to 0, 0.03, 0.06, 0.12, 0.23, and 0.46 mM of Se(IV) solution for 48 h. Afterwards, the roots were stained with NBT (E), Schiff's reagent (F), and Evan blue (G), respectively. *Asterisk* indicates that mean values of three replicates are significantly different between the treatments of Se(IV) and the control group (CK) (*P*<0.05).

Root tip is the important expansion zone for root elongation [50]. In order to test whether Se(IV) stress could induce ROS accumulation in root tips, we performed *in situ* detection of intracellular ROS generation by using specific fluorescent probe DCFH-DA. Compared to the control, the strong fluorescent density was observed in root tips in the presence of Se(IV) in a dose-dependent manner (Figure 2A, B). In a 24 h time-course experiment, treatment with 0.06 mM of Se(IV) resulted in the continuous accumulation of total ROS after 3 h of treatment (Figure 2C, D). The correlation analysis suggested that ROS generation was negatively correlated with root length under Se(IV) stress (Figure S1A).

Since O_2_^• **−**^ is one of the most important ROS produced by NADPH oxidase, the generation of O_2_^• **−**^ was detected histochemically using NBT. As expected, the generation of O_2_^• **−**^ in roots increased considerably with the increase in Se(IV) concentration (Figure 2E). Because the over-generation of ROS is closely related to the oxidative injury to plant cells, the oxidation of membrane lipids and the loss of plasma membrane integrity were determined using histochemical staining with Shiff's regent and Evans blue, respectively [28]. The roots of *B. rapa* treated with Se(IV) were stained extensively while the control group had only light staining (Figure 2F, G). These results confirmed the oxidative stress induced by Se(IV) in the roots of *B. rapa*.

### Inhibition of NADPH oxidase activity attenuated Se(IV)-induced oxidative injury and growth inhibition

**Figure 3:**
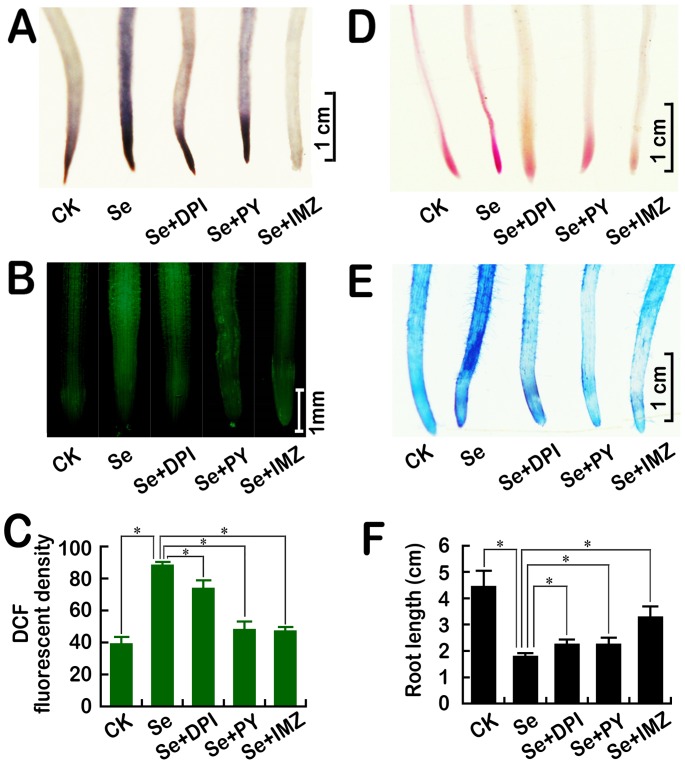
Effect of NADPH oxidase inhibitors (DPI, PY, and IMZ) on endogenous O_2_^• −^ (A), endogenous total ROS (B–C), lipid peroxidation (D), the loss of plasma membrane integrity (E), and the growth of roots under Se(IV) stress. The roots of seedlings were exposed to Se(IV) (0.06 mM), Se(IV) (0.06 mM)+DPI (10 µM), Se(IV) (0.06 mM)+PY (5 mM), and Se(IV) (0.06 mM)+IMZ (0.5 mM) for 48 h. Afterwards, the roots were stained with NBT (A), DCFH-DA (B), Schiff's reagent (D), and Evan blue (E), respectively. The density of DCF fluorescence were estimated (C). The roots of seedlings were exposed to the above treatment solutions for 72 h. Afterwards, the root length were measured (F). *Asterisk* indicates that mean values of three replicates are significantly different between the different treatments (*P*<0.05).

Three NADPH oxidase inhibitors (DPI, PY, and IM) were used to determine the involvement of NADPH oxidase in Se(IV)-induced oxidative stress and growth inhibition in roots. Compared to Se(IV) treatment alone, simultaneous treatment with NADPH oxidase inhibitors remarkably reduced the over-generation of O_2_^• **−**^ (Figure 3A) and the accumulation of total ROS (Figure 3B, C). In addition, blocking NADPH oxidase activity also alleviated Se-induced membrane lipids oxidation (Figure 3D), the loss of membrane integrity (Figure 3E), and growth inhibition (Figure 3F). These results suggested that Se(IV)-induced oxidative injury and growth inhibition resulted from NADPH oxidase-mediated over-generation of ROS.

### Se(IV) stress induced the expression of *Br_Rbohs* in roots

In order to investigate the effect of Se(IV) stress on the expression of *Rboh* genes coding NADPH oxidase, 12 *Br_Rboh* genes were identified from the genome of *B. rapa* by using *Rboh* homologues from Arabidopsis as baits for BLAST search. According to the sequence identity and phylogenetic relationship with other plant *Rbohs*, these 12 *Br_Rboh* genes were designated as *Br_RbohA* (Bra009266), *Br_RbohB* (Bra031658), *Br_RbohC* (Bra029194), *Br_RbohD* (Bra020724), *Br_RbohE1* (Bra025721), *Br_RbohE2* (Bra031070), *Br_RbohF* (Bra027764), *Br_RbohG1* (Bra019189), *Br_RbohG2* (Bra019191), *Br_RbohH* (Bra020270), *Br_RbohI* (Bra033151), and *Br_RbohJ* (Bra038274), respectively (Figure S2). The alignment of deduced amino acid sequences revealed that the obtained Br_Rbohs had many typically structural features of plant Rbohs (Figure S3). For instance, two Ca^2+^-binding EF-hand motifs are located in the N-terminal region of each Br_Rboh. The C-terminal regions of Br_Rbohs contain conserved FAD and NADPH binding sites. Two pairs of histidine residues indicated by blue box are conserved haem binding sites in both human and plant Rboh [51], [52].

**Figure 4:**
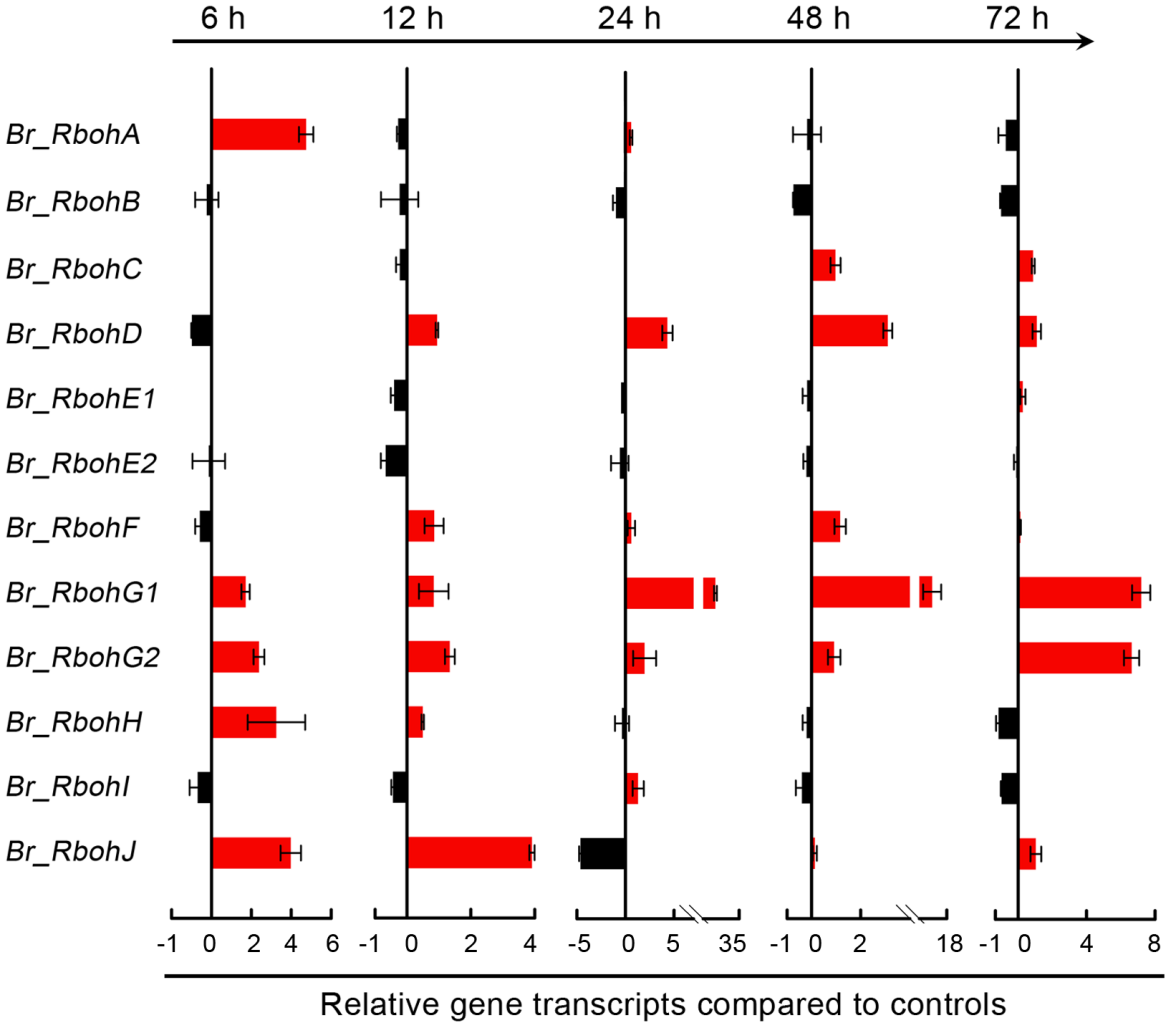
Effect of Se(IV) on the transcripts of *Br_RbohA-J*. The roots of seedlings were exposed to 0.06 mM of Se(IV) solution for 6, 12, 24, 48, and 72 h, respectively. The total RNA was extracted from roots for qRT-PCR analysis. *Actin* was used for cDNA normalization. The data were presented as the relative fold-change in transcript abundance of the target genes. *Red* column means up-regulation. *Black* column means down-regulation.

Next, the expression pattern of *Br_Rbohs* in roots was monitored under 0.06 mM of Se(IV) stress in a time-course experiment using qRT-PCR (Figure S4). The analysis of the fold-change of the relative transcript abundance suggested that most *Br_Rbohs* could be up-regulated in roots under Se(IV) treatment (Figure 4). Three *Br_Rbohs* (*A*, *H*, and *J*) were up-regulated in the early stage upon Se(IV) exposure (6–12 h) (Figure 4). However, the transcription of *Br_RbohC* was stimulated in the late stage upon Se(IV) exposure (48–72 h) (Figure 4). The transcription of four *Br_Rbohs* (*D*, *F*, *G1*, and *G2*) showed up-regulation through almost the whole tested period of Se(IV) exposure (Figure 4). These results indicated that NADPH oxidase-mediated over-generation of ROS might resulted from the up-regulation of *Br_Rbohs* in *B. rapa* under Se(IV) stress.

### Se(IV) stress stimulated the generation of endogenous NO in roots

**Figure 5:**
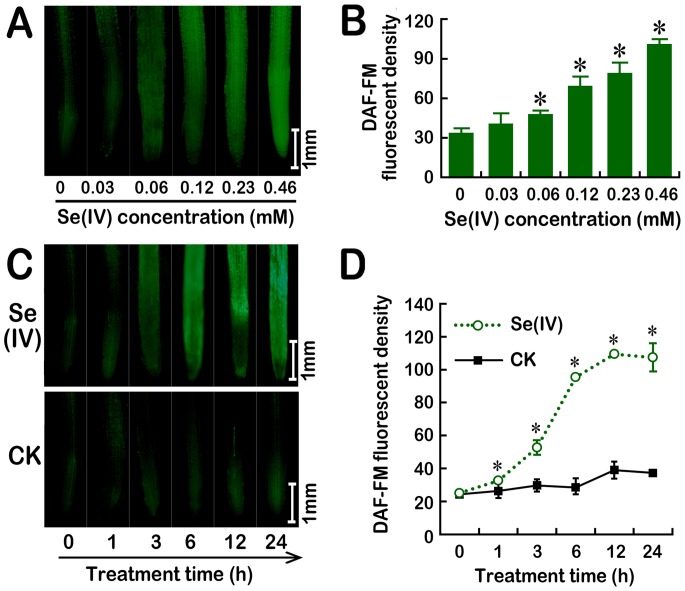
Effect of Se(IV) on the generation of endogenous NO in the roots of *B. rapa*. The roots of seedlings were exposed to 0, 0.03, 0.06, 0.12, 0.23, and 0.46 mM of Se(IV) solution for 48 h. Afterwards, the roots were loaded with DAF-FM DA for 15 min and immediately photographed (A). The relative DAF-FM fluorescent density in roots was estimated (B). (C–D) The image and density of DAF-FM fluorescence were obtained when the roots of seedlings were exposed to 0.06 mM of Se(IV) solution for 0, 1, 3, 6, 12, and 24 h, respectively.

The intracellular of NO was visually detected using NO specific fluorescent probe DAF-FM DA. Compared to the control, Se(IV) treatment stimulated the over-generation of endogenous NO in the root tips in a dose-dependent manner (Figure 5A, B). In a time-course experiment, treatment with 0.06 mM of Se(IV) resulted in the continuous accumulation of endogenous NO after treatment for 1–24 h (Figure 5C, D). The correlation analysis suggested that NO generation was negatively correlated with root length under Se(IV) stress (Figure S1B).

**Figure 6:**
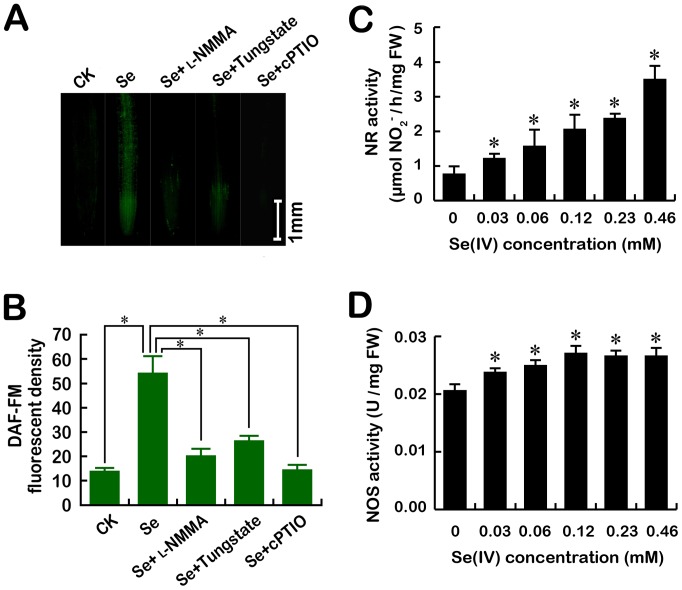
Identification of the sources of Se(IV)-induced NO generation in the roots of *B. rapa*. (A–B) The image and density of DAF-FM fluorescence were obtained when the roots of seedlings were exposed to Se(IV) (0.06 mM), Se(IV) (0.06 mM)+_L_-NMMA (200 µM), Se(IV) (0.06 mM)+Tungstate (30 µM), and Se(IV) (0.06 mM)+cPTIO (0.1 mM) for 48 h. *Asterisk* in (B) indicates that mean values of three replicates are significantly different between different treatments (*P*<0.05). The roots of seedlings were exposed to 0, 0.03, 0.06, 0.12, 0.23, and 0.46 mM of Se(IV) solution for 48 h. After that, NR activity (C) and NOS activity (D) in roots were measured, respectively. *Asterisk* in (C) and (D) indicates that mean values of three replicates are significantly different between Se(IV) treatment and the control (*P*<0.05).

To further ascertain the key enzyme responsible for endogenous NO generation induced by Se(IV), the effects of _L_-NMMA (NOS inhibitor) and tungstate (NR inhibitor) on the level of endogenous NO in the roots of *B. rapa* under Se(IV) exposure were evaluated. Compared to the control, the content of NO increased considerably under Se(IV) treatment. However, both Se(IV)+_L_-NMMA and Se(IV)+tungstate significantly reduced the NO abundance as compared to the treatment of Se(IV) alone (Figure 6A, B). The addition of cPTIO, a NO scavenger, could decrease the over-generation of NO induced by Se(IV) as well. The enzymatic assay indicated that treatment with Se(IV) induced the increases in NR and NOS activity as compared to their control groups, respectively (Figure 6C, D). These results suggested that Se(IV) exposure could induce the rapid accumulation of endogenous NO through both NOS- and NR-dependent pathway.

### Blocking endogenous NO generation reduced Se(IV)-induced ROS accumulation and the expression of *Br_Rbohs* in roots

**Figure 7:**
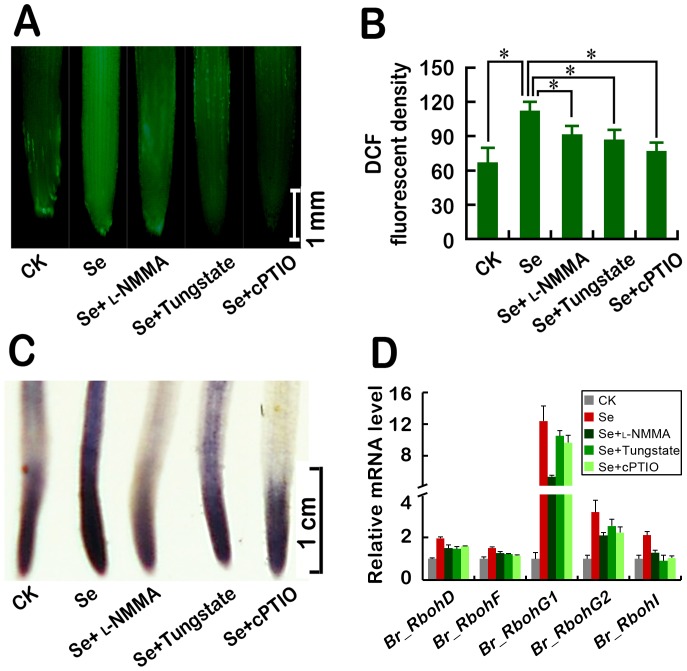
Effects of NO synthesis inhibitors (_L_-NMMA and tungstate) and NO scavenger (cPTIO) on the generation of endogenous ROS (A–B), O_2_^• −^ (C), and the expression of several *Br_Rbohs* (D) in Se(IV)-treated roots of *B. rapa*. The roots of seedlings were exposed to Se(IV) (0.06 mM), Se(IV) (0.06 mM)+_L_-NMMA (200 µM), Se(IV) (0.06 mM)+Tungstate (30 µM), and Se(IV) (0.06 mM)+cPTIO (0.1 mM) for 72 h. The roots were loaded with DCFH-DA for 15 min and immediately photographed (A). The relative DCF fluorescent density in roots was estimated (B). *Asterisk* indicates that mean values of three replicates are significantly different between the different treatments (*P*<0.05). The roots were stained with NBT for the detection of O_2_^• **−**^ (C). The total RNA was extracted from roots under the above treatments for 24 h for qRT-PCR analysis of the transcripts of *Br_RbohD*, *F*, *G1*, *G2*, and *I*. *Actin* was used for cDNA normalization (D).

The correlation analysis suggested that NO generation was postively correlated with ROS accumulation in roots under Se(IV) stress (Figure S1C). In order to verify whether NO is involved in Se(IV)-induced ROS accumulation, the abundance of endogenous ROS in roots was determined when Se(IV)-induced NO generation was scavenged using cPTIO or blocked using _L_-NMMA and tungstate. The content of total ROS indicated DCF fluorescence increased remarkably upon exposure to 0.06 mM of Se(IV), while the addition of _L_-NMMA, tungstate, and cPTIO could decrease the accumulation of ROS in roots, respectively (Figure 7A, B). Both _L_-NMMA and tungstate could prohibited Se(IV)-induced the over-generation of O_2_^• **−**^ by NBT staining indicated by as well (Figure 7C). The analysis of the transcripts of *Br_Rbohs* showed that _L_-NMMA and tungstate could inhibit Se(IV)-induced increase in the expression of *Br_RbohD*, *F*, *G1*, *G2*, and *I* (Figure 7D), which suggested that both NOS- and NR-dependent NO generation were responsible for Se(IV)-induced ROS accumulation by regulating the expression of several *Br_Rbohs* in roots.

### Analysis of the nitric oxide-responsive cis-elements in the promoters of *Br_Rbohs*

According to the identification of NO-responsive *cis*-elements (NREs) from higher plants [53], [54], several NREs (e.g. ACGT Box, MYCL, and W-BOX) were obtained from the promoter region of tomato *Br_RbohD*, *F*, *G1*, *G2*, and *I* (Table S2). This result suggested that NO induced the expression of *Br_Rbohs* by possibly regulating these NREs in their promoters.

### Blocking endogenous NO generation attenuated Se(IV)-induced oxidative injury and growth inhibition in roots

**Figure 8:**
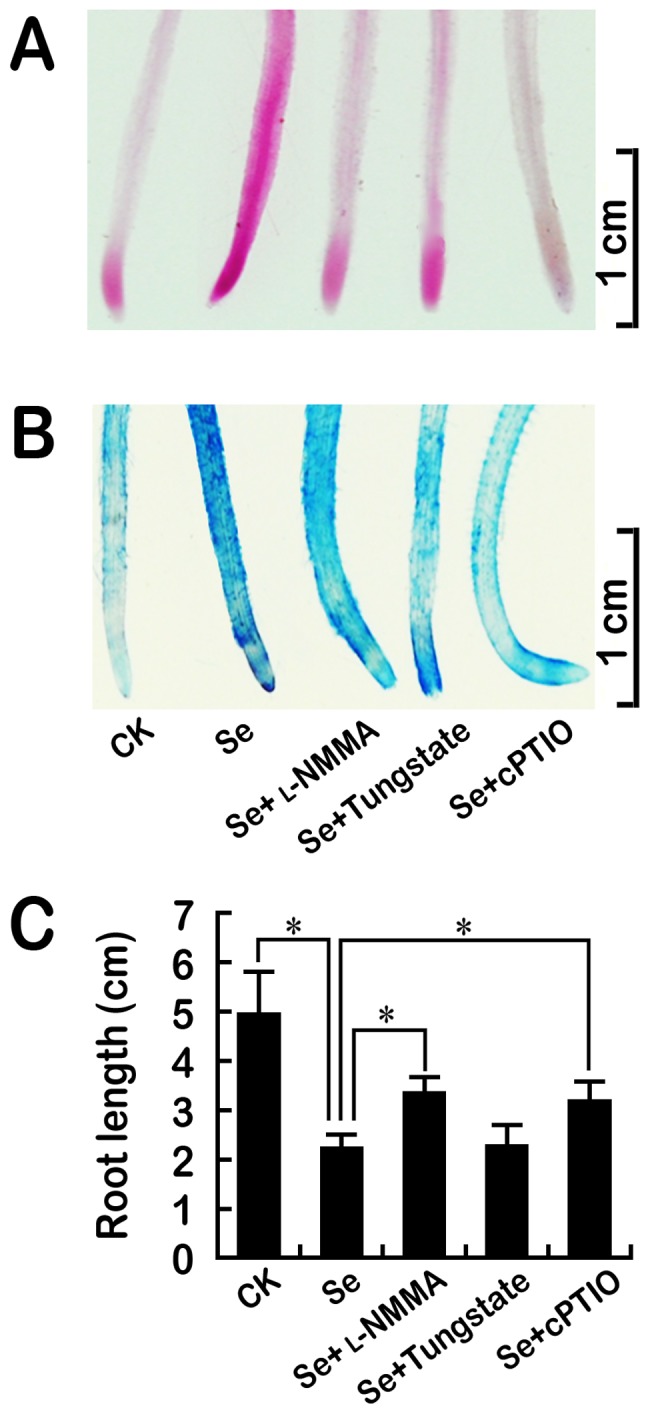
Effects of NO synthesis inhibitors (_L_-NMMA and tungstate) and NO scavenger (cPTIO) on lipid peroxidation (A), the loss of plasma membrane integrity (B), and root elongation under Se(IV) stress. The roots of seedlings were exposed to Se(IV) (0.06 mM), Se(IV) (0.06 mM)+_L_-NMMA (200 µM), Se(IV) (0.06 mM)+Tungstate (30 µM), and Se(IV) (0.06 mM)+cPTIO (0.1 mM) for 72 h. Afterwards, the roots were stained with Schiff's reagent (A) and Evan blue (B), respectively. The root length was measured as well (C). *Asterisk* indicates that mean values of three replicates are significantly different between the different treatments (*P*<0.05).

To obtain more solid evidence for the involvement of NO in Se(IV)-induced phytotoxicity, we determined the effect of the decrease in NO generation on Se(IV)-induced oxidative injury. The lipid peroxidation and loss of membrane integrity induced by Se(IV) could be attenuated by the addition of _L_-NMMA, tungstate, and cPTIO, respectively (Figure 8A, B). Se(IV)-inhibited root growth could be significantly recovered by the addition of _L_-NMMA and cPTIO, respectively (Figure 8C).

### Treatment with SNP aggravated Se(IV)-induced phytotoxicity

**Figure 9:**
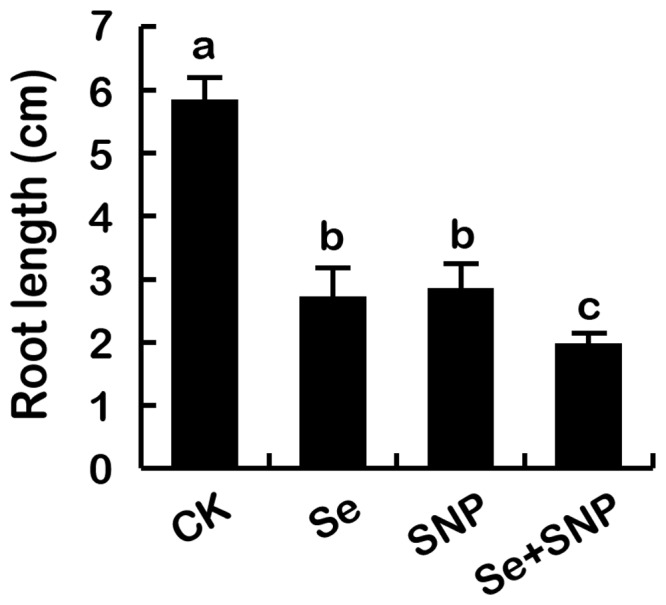
Effect of SNP on the root growth under Se(IV) stress. The roots of seedlings were exposed to Se(IV) (0.06 mM), SNP (250 mM), and Se(IV) (0.06 mM)+SNP (250 mM) for 72 h. After that, the length of roots were measured. The mean values of three replicates followed by the same letter were not significantly different at *P*<0.05.

SNP is a frequently used NO donor. Treatment with SNP alone showed similar inhibitory effect with Se(IV) on root growth. Compared to the Se(IV) treatment alone, simultaneous treatment with Se(IV) and SNP resulted in more inhibitory effect on root growth (Figure 9).

## Discussion

Previous studies have demonstrated that Se modifies various physiological process in plants [55], [56]. The major adverse effect of Se on plants is the suppression of growth and induction of oxidative stress [16], [17], [57]. Recently, NO is shown to be a possible mediator of oxidative stress in plants [41]. Whether and how NO triggers heavy metal-induced oxidative stress in plants is little known. In the present study, four lines of evidence indicated that both NOS- and NR-dependent NO generation mediated Se(IV)-induced oxidative stress and growth stunt in the roots of *B. rapa* by regulating a group of *Br_Rbohs* coding for NADPH oxidase. First, Se(IV)-induced ROS accumulation, oxidative stress, and growth inhibition were NADPH oxidase-dependent. Second, the expression of most *Br_Rboh* homologues were up-regulated in *B. rapa* under Se(IV) stress. Third, Se(IV) stimulated both NOS- and NR-dependent generation of endogenous NO. Fourth, blocking NOS- and NR-dependent NO generation reduced Se(IV)-induced ROS accumulation, which further resulted in the alleviation of Se(IV)-induced oxidative injury and growth stunt.

The suppression of plant endogenous NO induced by heavy metals has been extensively reported [58], but the accumulation of NO may also be linked to heavy metal-induced toxicity in plants [34], [35], [59]. In a recent study, Al-induced growth inhibition of wheat root was dependent on the accumulation of endogenous NO [38]. In our present study, Se(IV) stress induced rapid accumulation of endogenous NO in both NR- and NOS-dependent pathway, which was closely associated with growth inhibition of roots. Besides of NR and NOS, polyamine is suggested to be a potential trigger of NO generation in plants [60]. Since heavy metals and other abiotic stresses (e.g. salt and osmosis) can induce the biosynthesis of polyamine [61], thus it is of interest to further investigate whether and how Se(IV) induces polyamine-based NO generation.

Se(IV) stress induced faster accumulation of NO than that of ROS. The removal of endogenous NO could significantly attenuated Se(IV)-induced ROS accumulation and oxidative injury, suggesting that the ROS accumulation and the following oxidative injury were dependent on the rapid accumulation of endogenous NO in the roots of *B. rapa* under Se(IV) stress. Thus, we established a positive link between NO accumulation and oxidative injury in plants under Se(IV) stress. However, Se(IV) stress can inhibit endogenous NO generation in the root of *Arabidopsis*, which further impacted plant auxin signalling [62]. Therefore, NO seems to play dual roles in regulating plant adaptions upon Se(IV) stress by modulating either hormone signalling or oxidative stress, which may be involved complicated regulatory mechanisms that needs to be illuminated further.

Plant NADPH oxidases have been considered as one of the important enzymatic complexes to deliberately generate ROS [63]. In the present study, pharmacological experiment suggested that Se(IV) induced oxidative injury in *B. rapa* through NADPH oxidase-dependent ROS accumulation. Se(IV)-induced root inhibition could be partially recovered by the addition of NADPH oxidase inhibitors, suggesting that NADPH oxidase-dependent ROS accumulation contributed to Se toxicity. *RbohD* has been closely linked to ROS generation in plants under copper (Cu), Cd, salt, and wound stress [64]–[67]. In *Zea mays*, abscisic acid (ABA)-triggered ROS generation has been linked to the *RbohA-D* [51]. *RbohF*-dependent ROS generation has been associated with pathogen recognition in multiple plant species [63]. In the present study, the induction of *Br_Rbohs* expression by NO under Se(IV) stress may resulted from the presence of NREs in the promoter regions of *Br_Rbohs*. The differential regulation of plant *Rbohs* by Se(IV) and other environmental stimuli may results from the diversity of upstream regulators of *Rbohs*.

In mammals, NO tends to lower the expression of *Nox1/2*, homologues of *Rboh* in human cells, leading to the inhibition of ROS production [68], [69]. Our present study found the opposite results that NO could stimulate the expression of *Br_Rbohs*, leading to the over-generation of ROS in *B. rapa* under Se(IV) stress. Heavy metal-modulated phosphatidic acid (PA) and mitogen-activated protein kinase (MAPK) signalling cassettes have been extensively reported in plants [70], [71]. PA and ABA are the well-characterized upstream regulators of *RbohD* and *RobhF* in *Arabidopsis* [72], [73]. In pathogen defensive responses, NO induces ROS burst through PA signalling in plants [74]. In *Z. mays*, ABA-induced NO generation is indispensable for the activation of MAPK5 that positively regulates the expression of *Zmrbohs* for ROS production [51], [75]. Thus, whether Se(IV)-triggered NO generation regulates the expression of *Br_Rbohs* through PA or MAPK signalling remains to be further illuminated.

## Conclusions

The anthropogenic release of excessive Se gives rise to the increase in Se concentration in agricultural environment, which further shows adverse effect on crop growth. Here, we demonstrate that Se(IV)-induced oxidative injury resulted from NADPH oxidase-dependent ROS generation contributes to the growth inhibition of *B. rapa* roots. Endogenous NO seems to stimulate ROS generation by stimulating the expression of several *Br_Rbohs* coding for NADPH oxidase. These results not only propose the toxic role of NO in mediating heavy metal-induced oxidative stress, but also shed new light on the investigation of Se(IV)-induced phytotoxicity. Further identification of plant responses to Se stress will be helpful for crop breeding designed to improve plant tolerance to Se-contaminated environment.

## Supporting Information

Figure S1 **Correlation analysis among root growth, ROS accumulation, and NO accumulation in roots under Se(IV) stress at 0, 0.03, 0.06, 0.12, 0.23, and 0.46 mM.** (A) Correlation between root length and ROS accumulation indicated by DCF fluorescence. (B) Correlation between root length and NO accumulation indicated by DAF-FM fluorescence. (C) Correlation between ROS accumulation indicated by DCF fluorescence and NO accumulation indicated by DAF-FM fluorescence.
(TIF)

Figure S2 **The phylogenetic relationship of Br_RbohA-J and their related members of Rboh family.** Species name: Aet, *Aegilops tauschii*; At, *Arabidopsis thaliana*, Bo, *Brassica oleracea*; Br, *Brassica rapa*; Ls, *Lepidium sativum*; Nt, *Nicotiana tabacum*; OsI, *Oryza sativa* Indica Group; OsJ, *Oryza sativa* Japonica Group; St, *Solanum tuberosum*; Tu, *Triticum urartu*.
(TIF)

Figure S3 **Alignment of the predicted amino acid sequences of Br_RbohA-J.** Multiple alignment of predicted Br_RbohA-J protein was made with Clustal Mega. *Dark shading* and *dull grey shading* reveal 100% and 75% sequence conservation, respectively. The *red line* indicates the conserved functional domains of NADPH oxidase, such as EF hand I and II, FAD-isoalloxazine site, NADPH-ribose site, and NADPH-adenine site. In two EF hands, dashes indicate variable amino acid residues. X, Y, Z, and –X, contain oxygen within their side chains. –Z is usually glutamic acid. *Blue box* indicates histidine residues involved in haem binding.
(TIF)

Figure S4 **Effect of Se(IV) on the transcripts of** ***Br_RbohA-J*** **.** The roots of seedlings were exposed to 0.06 mM of Se(IV) solution for 6, 12, 24, 48, and 72 h, respectively. The total RNA was extracted from roots for qRT-PCR analysis. *Actin* was used for cDNA normalization.
(TIF)

Table S1 **Sequences of oligonucleotide primers for qRT-PCR.** F: forward; R: reverse.
(DOCX)

Table S2 **Distribution of NO-responsive** ***cis*** **-elements (AGCT Box, MYCL, and W-BOX) in the promote region of** ***Br_RbohD*** **,** ***F*** **,** ***G1*** **,** ***G2*** **, and** ***I*** **in** ***B. rapa*** **.** The plant motifs were predicted based on the publicly available *cis*-acting regulatory elements database PLACE (http://bioinformatics.psb.ugent.be/webtools/plantcare/html/) as a reference. Forward sequence is indicated as (+) while the complementary sequence is indicated as (−).
(DOCX)
