# Supplementary figures and images for: The Endogenous Nitric Oxide Mediates Selenium-Induced Phytotoxicity by Promoting ROS Generation in Brassica rapa
Source: PLoS One. 2014 Oct 21;9(10):e110901. doi: 10.1371/journal.pone.0110901 (PMC4204988; doi:10.1371/journal.pone.0110901)

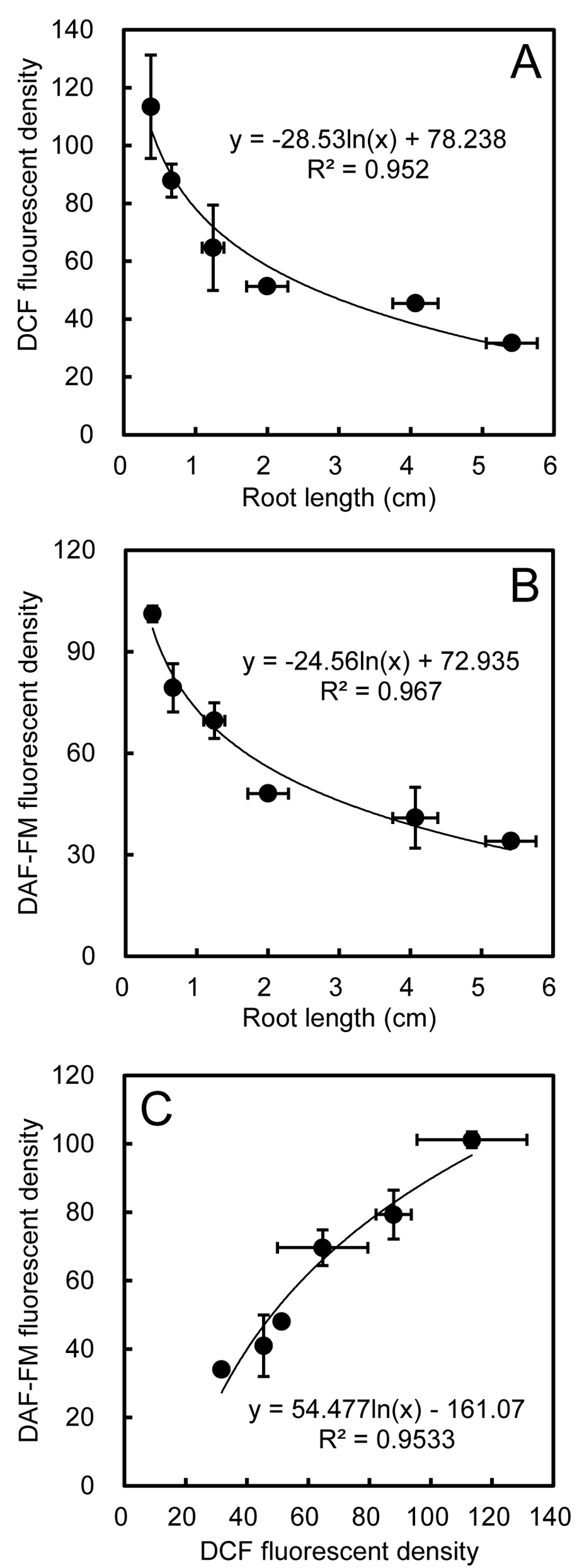

Supplement: Figure S1 — Correlation analysis among root growth, ROS accumulation, and NO accumulation in roots under Se(IV) stress at 0, 0.03, 0.06, 0.12, 0.23, and 0.46 mM. (A) Correlation between root length and ROS accumulation indicated by DCF fluorescence. (B) Correlation between root length and NO accumulation indicated by DAF-FM fluorescence. (C) Correlation between ROS accumulation indicated by DCF fluorescence and NO accumulation indicated by DAF-FM fluorescence. (TIF) [file pone.0110901.s001.tif]

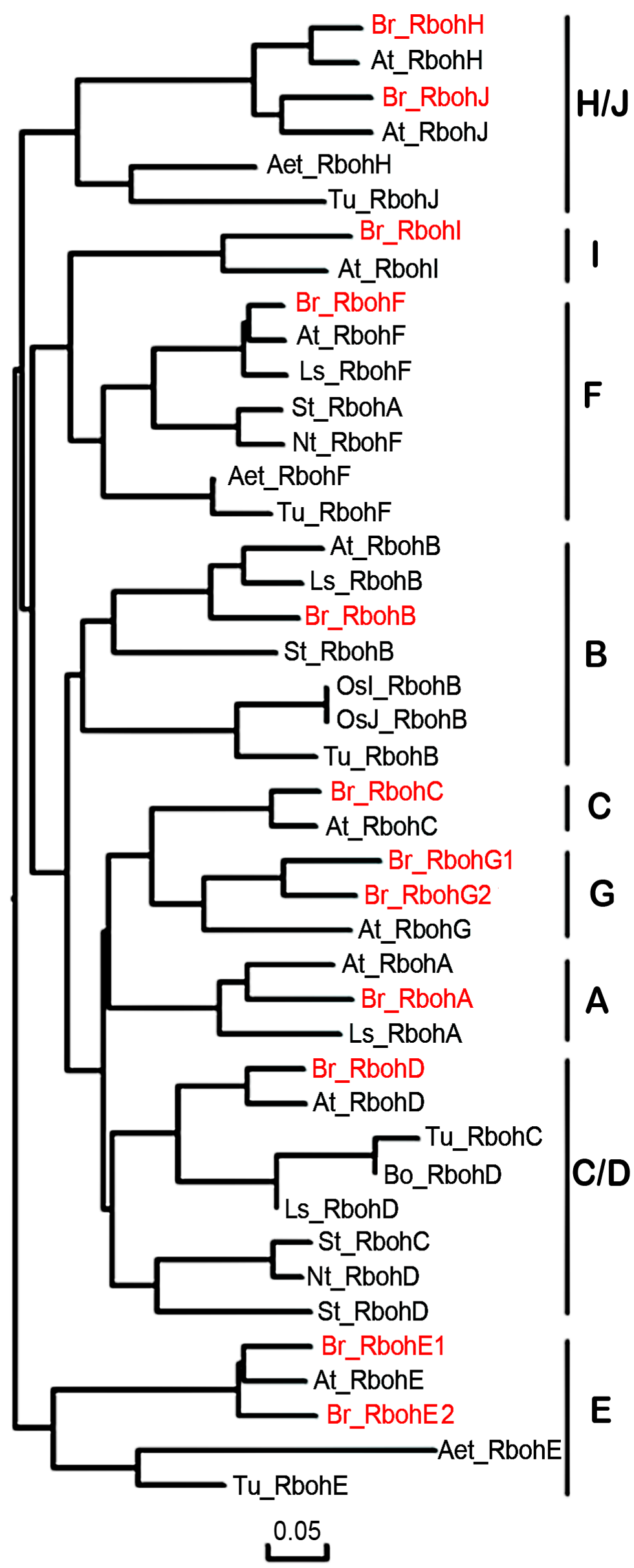

Supplement: Figure S2 — The phylogenetic relationship of Br_RbohA-J and their related members of Rboh family. Species name: Aet, Aegilops tauschii; At, Arabidopsis thaliana, Bo, Brassica oleracea; Br, Brassica rapa; Ls, Lepidium sativum; Nt, Nicotiana tabacum; OsI, Oryza sativa Indica Group; OsJ, Oryza sativa Japonica Group; St, Solanum tuberosum; Tu, Triticum urartu. (TIF) [file pone.0110901.s002.tif]

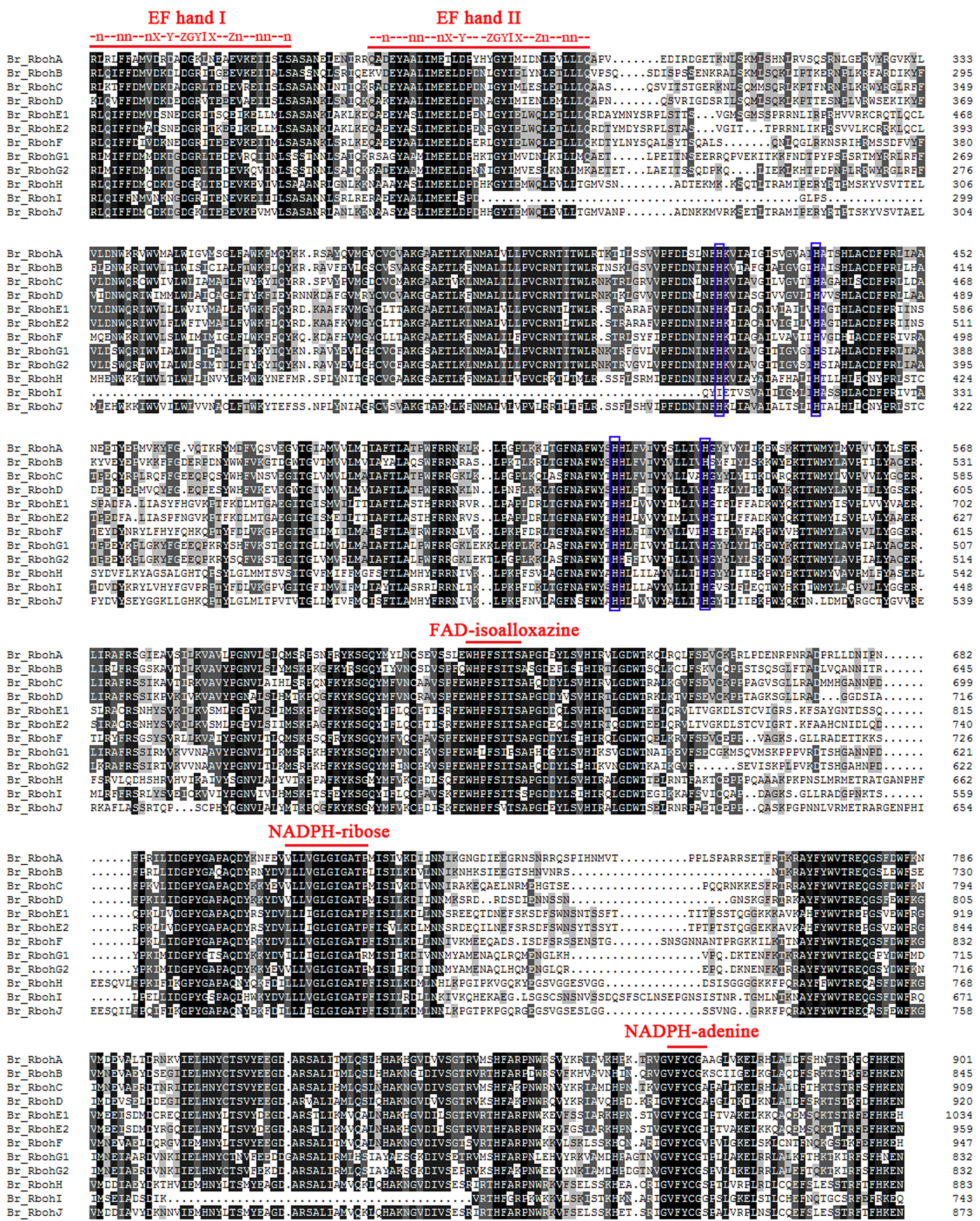

Supplement: Figure S3 — Alignment of the predicted amino acid sequences of Br_RbohA-J. Multiple alignment of predicted Br_RbohA-J protein was made with Clustal Mega. Dark shading and dull grey shading reveal 100% and 75% sequence conservation, respectively. The red line indicates the conserved functional domains of NADPH oxidase, such as EF hand I and II, FAD-isoalloxazine site, NADPH-ribose site, and NADPH-adenine site. In two EF hands, dashes indicate variable amino acid residues. X, Y, Z, and –X, contain oxygen within their side chains. –Z is usually glutamic acid. Blue box indicates histidine residues involved in haem binding. (TIF) [file pone.0110901.s003.tif]

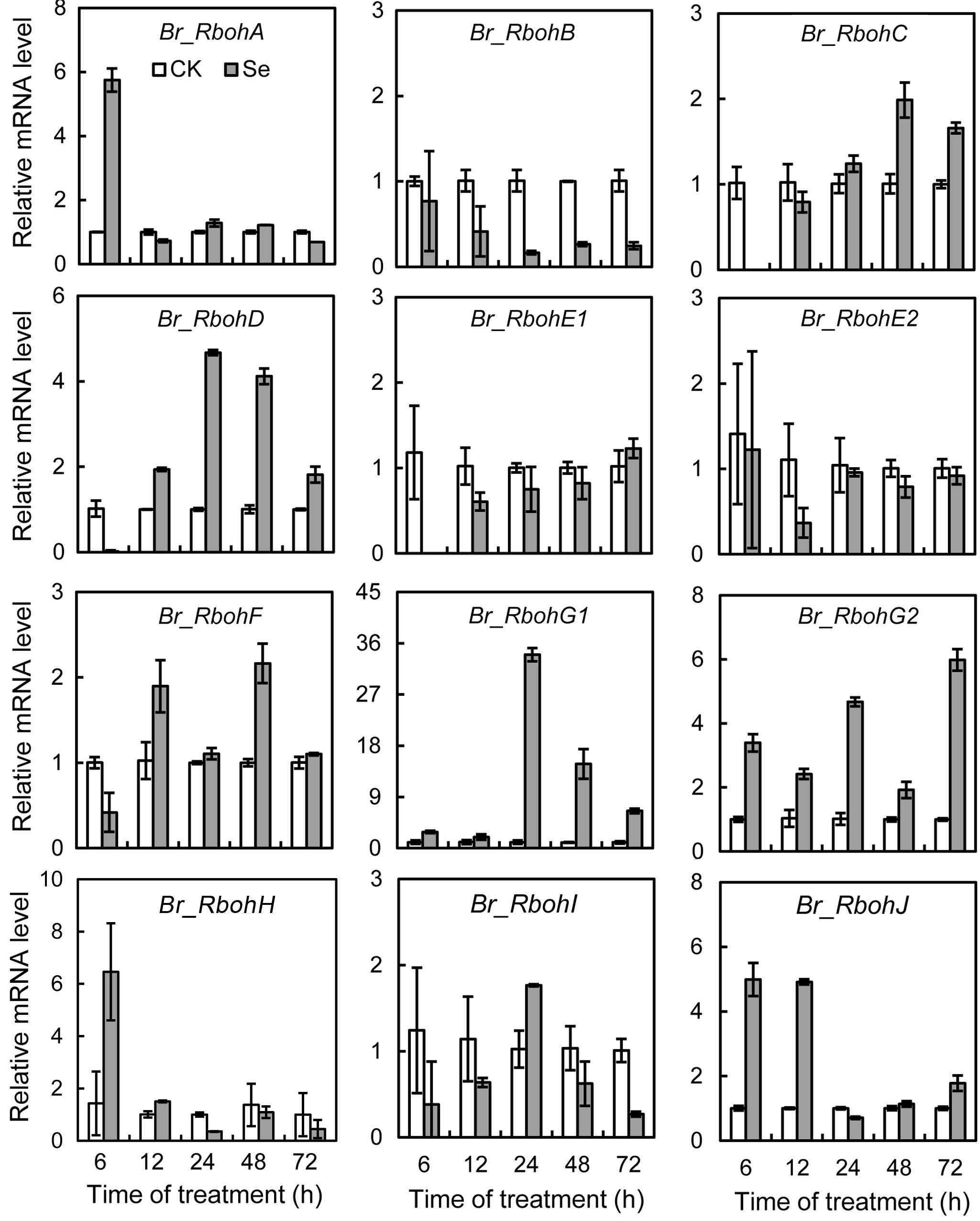

Supplement: Figure S4 — Effect of Se(IV) on the transcripts of Br_RbohA-J . The roots of seedlings were exposed to 0.06 mM of Se(IV) solution for 6, 12, 24, 48, and 72 h, respectively. The total RNA was extracted from roots for qRT-PCR analysis. Actin was used for cDNA normalization. (TIF) [file pone.0110901.s004.tif]
