# Supplementary material for: The Endogenous Nitric Oxide Mediates Selenium-Induced Phytotoxicity by Promoting ROS Generation in Brassica rapa
Source: PLoS One. 2014 Oct 21;9(10):e110901. doi: 10.1371/journal.pone.0110901 (PMC4204988; doi:10.1371/journal.pone.0110901)
Supplement: Table S1 — Sequences of oligonucleotide primers for qRT-PCR. F: forward; R: reverse. (DOCX) [file pone.0110901.s005.docx]

**Table S1.** Sequences of oligonucleotide primers for qRT-PCR. F: forward; R: reverse.

| **Name of gene** |  | **Primer sequence (5'-3')** |
| --- | --- | --- |
| *Br_RbohA* | **F** | CCCCAGCAAGAAGAAGTGAG |
|  | **R** | TCGTGATCAGTGCAGACCTC |
| *Br_RbohB* | **F** | CAGCAAGATGTTGAGCCAGA |
|  | **R** | AACCCAAGACCTCAAACACG |
| *Br_RbohC* | **F** | TGCTGCTGTTTCTCCATTTG |
|  | **R** | TGCATCATGTCGGCTCTAAG |
| *Br_RbohD* | **F** | GGGGATAATGGACGAGGTTT |
|  | **R** | ATTCTTTTGTCGGGATGCTG |
| *Br_RbohE1* | **F** | GGCTACCTGCACCACTTGAT |
|  | **R** | GGACCTACAAGCTCGCAGAC |
| *Br_RbohE2* | **F** | GTCTACCGGCACCACTTGAT |
|  | **R** | CACCAGAGGAACCGAGATGT |
| *Br_RbohF* | **F** | GAACAGCACAGGAAGCAACA |
|  | **R** | AGCTCTGCCACTTCGTTCAT |
| *Br_RbohG1* | **F** | ACTGATCGGTCTTGGGATTG |
|  | **R** | TGCGATTTCGTTCATGATGT |
| *Br_RbohG2* | **F** | GGTGCACCAGCACAAGACTA |
|  | **R** | TCCCTTTGCAATCCATTCTC |
| *Br_RbohH* | **F** | GGCATCCATTCTCCATCACT |
|  | **R** | TCTTCAAAGTGGGGATTTGC |
| *Br_RbohI* | **F** | TCATGAGCGAAATTGCTGAC |
|  | **R** | GTTCTTTGCGGAACTCGAAC |
| *Br_RbohJ* | **F** | ACATGGCTTTGGTCTTGGTC |
|  | **R** | AGCAAGTGTAGTGCCGTGTG |
| *Actin* | **F** | CTATCCTCCGTCTCGATCTCGC |
|  | **R** | CTTAGCCGTCTCCAGCTCTTGC |
