# Supplementary material for: The Endogenous Nitric Oxide Mediates Selenium-Induced Phytotoxicity by Promoting ROS Generation in Brassica rapa
Source: PLoS One. 2014 Oct 21;9(10):e110901. doi: 10.1371/journal.pone.0110901 (PMC4204988; doi:10.1371/journal.pone.0110901)
Supplement: Table S2 — Distribution of NO-responsive cis -elements (AGCT Box, MYCL, and W-BOX) in the promote region of Br_RbohD , F , G1 , G2 , and I in B. rapa . The plant motifs were predicted based on the publicly available cis-acting regulatory elements database PLACE (http://bioinformatics.psb.ugent.be/webtools/plantcare/html/) as a reference. Forward sequence is indicated as (+) while the complementary sequence is indicated as (−). (DOCX) [file pone.0110901.s006.docx]

**Table S2.** Distribution of NO-responsive *cis*-elements (AGCT Box, MYCL, and W-BOX) in the promote region of *Br_RbohD*, *F*, *G1*, *G2*, and *I* in *B. rapa*. The plant motifs were predicted based on the publicly available *cis*-acting regulatory elements database PLACE (<http://bioinformatics.psb.ugent.be/webtools/plantcare/html/>) as a reference. Forward sequence is indicated as (+) while the complementary sequence is indicated as (-).

|  | ***Br_RbohD*** | ***Br_RbohF*** | ***Br_RbohG1*** | ***Br_RbohG2*** | ***Br_RbohI*** |
| --- | --- | --- | --- | --- | --- |
| **ACGT Box**  **(ACGT)** | 96 (+)  134 (+)  748 (+)  913 (+)  1299 (+)  1907 (+)  96 (-)  134 (-)  748 (-)  913 (-)  1299 (-)  1907 (-) | 70 (+)  309 (+)  1223 (+)  1705 (+)  1942 (+)  70 (-)  309 (-)  1223 (-)  1705 (-)  1942 (-) | 250 (+)  436 (+)  759 (+)  927 (+)  1222 (+)  1330 (+)  1661 (+)  1677 (+)  1765 (+)  1852 (+)  250 (-)  436 (-)  759 (-)  927 (-)  1222 (-)  1330 (-)  1661 (-)  1677 (-)  1765 (-)  1852 (-) | 211 (+)  417 (+)  521 (+)  959 (+)  1727 (+)  1883 (+)  1971 (+)  211 (-)  417 (-)  521 (-)  959 (-)  1727 (-)  1883 (-)  1971 (-) | 460 (+)  1089 (+)  460 (-)  1089 (-) |
| **MYCL**  **(CANNTG)** | 397 (+)  703 (+)  1605 (+)  397 (-)  703 (-)  1605 (-) | 298 (+)  573 (+)  974 (+)  1477 (+)  298 (-)  573 (-)  974 (-)  1477 (-) | 218 (+)  640 (+)  782 (+)  873 (+)  970 (+)  1021 (+)  1817 (+)  218 (-)  640 (-)  782 (-)  873 (-)  970 (-)  1021 (-)  1817 (-) | 383 (+)  1640 (+)  383 (-)  1640 (-) | 383 (+)  705 (+)  754 (+)  839 (+)  1770 (+)  383 (-)  705 (-)  754 (-)  839 (-)  1770 (-) |
| **W-BOX**  **[TTGAC(/T)]** | 137 (+)  1263 (+)  1645 (+)  1676 (+)  1692 (+)  1944 (+)  1984 (+)  1295 (-) | 415 (+)  786 (+)  386 (-)  1391 (-)  1701 (-) | 190 (+) | 715 (-)  1127 (-)  1939 (-) | 383 (+)  705 (+)  754 (+)  839 (+)  1770 (+)  383 (-)  705 (-)  754 (-)  839 (-)  1770 (-) |
